# Child-centered home service design for a family robot companion

**DOI:** 10.3389/frobt.2024.1346257

**Published:** 2024-07-29

**Authors:** Hyo Jeong Lym, Hyo In Son, Da-Young Kim, Juhyun Kim, Min-Gyu Kim, Jae Hee Chung

**Affiliations:** ^1^ Human-Robot Interaction Center, Korea Institute of Robotics and Technology Convergence, Pohang, Republic of Korea; ^2^ Industrial Design Major, Graduate School of Design, University of Hongik, Seoul, Republic of Korea; ^3^ Service Design Major, Graduate School of Industrial Arts, University of Hongik, Seoul, Republic of Korea

**Keywords:** human-robot interaction, child-robot interaction, social robot, service design, interaction design, childcare

## Abstract

The home robot-based child activity service aims to cultivate children’s social emotions. A design theme was produced by interviewing child development experts and parents. The activity service is composed of 50 plays and 70 conversations. These were developed based on activities from psychomotor therapy and the guidelines of Ministry of Early Childhood Education in South Korea. In the field test, 50 children aged five–seven years participated to experience the activity services at home for 4 days. After completing the 4 days of field testing, we conducted customer satisfaction (CSAT) surveys, Godspeed evaluations and interviews to quantitatively and qualitatively verify the evaluations by the children and parents. As a result, 92% of the children and 80% of the parents evaluated that they were satisfied with the service. In addition, our results revealed that the social robot-based service contributed to improving the relationship between children and families by functioning as a messenger. Finally, the lessons learned from the service development and field tests were discussed to aid service designers and robotics engineers.

## 1 Introduction

With the weakening of social bonds owing to the COVID-19 incident, children’s face-to-face activities have decreased. This has resulted in increased mental health issues of children, such as anxiety, despair, and aggression (Colizzi et al., 2020; Duan et al., 2020; Yeasmin et al., 2020). Children’s social and emotional immaturity is likely to develop into interpersonal problems (Hay et al., 2004) and social nonadaptation (Richard and Dodge, 1982). Hence, they need appropriate and timely help. Professional social–emotional development services provided by social robots can help children develop social emotions. we designed a home robot-based child activity service that aims to cultivate children’s social emotions.

Many prior studies have stated that services provided by social robots can help children develop their emotions (Freitas et al., 2017; Rakhymbayeva et al., 2021; Nature Machine Intelligence, 2023). Studies have demonstrated that children’s social anxiety reduce, and joyful feelings improve with the participation of social robots; physical activities such as aerobics, dance, and soccer; and conversations through LLM-based live chat (Podpečan, 2023). Game-based emotional cognitive activities conducted by social robots help children develop empathy and enhance emotional intelligence (Rafique et al., 2020). Role-playing with robots is known to help children learn the skills of identifying and expressing others’ emotions effectively (Leite et al., 2017). Based on the results of previous studies indicating that various activities with robots affect children’s development, the social robot service of this study was developed focusing on play and conversation activities.

Maintaining a positive relationship between children and robots is important for the continued service of social robots. The first process of building positive relationships between social robots and children is rapport formation (Crossman et al., 2018). Rapport implies positive ties between individuals (Kory et al., 2019). Children develop more empathy and connections with robots that have names and backgrounds than with those that do not (Darling et al., 2015). Therefore, it is effective for a robot to have unique storytelling (name, background) to form better rapport with children. In addition, the self-disclosure by robots plays an important role in building relationships with children (Ligthart et al., 2019). Children tend to share a substantial amount of personal feelings or true stories with their peers with whom they have formed good rapport (Rotenberg and Mann, 1986). Therefore, to continuously understand the child’s condition through social robots and help social and emotional development, the rapport between the robot and child needs to be effective.

After positive rapport has been formed, a method is required to maintain the interaction between children and robots. Name-calling, vocal answers, movement language, and physical touch are methods to increase the intimacy between the robot and child. When a robot calls children’s names, they realize that it knows them well. This increases their intimacy with the robots (Kanda et al., 2007). Furthermore, robots’ courteous responses and attitudes enhance children’s favorability toward robots (Lee et al., 2021). Robots may express their emotions through movement language. This enables these to respond to children’s reactions with positivity, comprehension, praise, and empathy (Beazeal, et al., 2005). Physical touch such as stroking and hugging directly increases intimacy. This makes robots to be perceived as closer to humans and have a positive impact on long-term friendship and trust (Cramer et al., 2009; Nie et al., 2012). Based on previous studies, this study was designed to form and maintain positive interactions between social robots and children.

The study aimed to 1) develop home robot-based activities that promote the development of children’s social emotions and 2) verify their effectiveness. 3) It also developed a robot–child interaction method to contribute to the development of the field of robot–child interaction research. The home robot-based children’s activity service developed in this study includes three important aspects. According to the parent interview, parents have limitations in the approach toward playing with their children and are inquisitive regarding other approaches to playing. They stated their intention to identify approaches to playing using everyday articles at home. The activities for children and robot were developed based on various methods used in psychomotor therapy. Second, social robots were developed to function as a peer for children. Social robots would develop with children and exist as the only objects designed for children to excite their interest (“Big bro”). Third, social robots and children’s conversations improved family communication. Parent interviews revealed that through robots, parents identified thoughts and experiences of their children that they were earlier unaware of. Therefore, the robot’s identity was designed as family messengers to enhance understanding between children and their families. This helped improve family relationships.

This paper is structured as follows: In Chapter 2, the needs of child education experts and parents are identified. Moreover, a home service design based on a psychomotor approach is presented. The design of the interaction between children and social robots is detailed in Chapter 3. Chapter 4 addresses the empirical design and approach and evaluates the experimental data. Finally, Chapter 5 discusses the contribution and importance of this study.

## 2 Design of home robot-based activity service for children

### 2.1 In-depth interview

To develop a robot-based home service, we conducted in-depth interviews with six child development and care experts and six parents of children aged seven–nine to identify the requirements. Owing to the COVID-19 pandemic, participants could opt between face-to-face and online interviews. The interviews were conducted by asking child development experts regarding the facility’s offerings, Therapy technique, and limits. Child development experts typically provide treatment through one-on-one play. The therapy technique is to enable children to learn on their own through indirect coaching. Child development experts state that even if a child is shy or introverted, the child’s voluntary participation may increase if he or she waits for the child to speak first. Finally, child development experts are concerned that parents have insufficient knowledge of professional treatment, which makes it difficult to sustain the treatment effect in the facility.

Additionally, parents were interviewed regarding the difficulty of raising children at home and the experience of playing with their children. The opinions of parents varied. First, parents wished to be informed regarding various play activities. The range of play they were aware of was limited. Parents wished to conduct play activities using materials that can be accessed conveniently in everyday life. Second, parents wished to be better informed regarding their children according to their gender and personality more deeply. Third, parents wished to be informed regarding the relationship of their children with their friends, their external social life experiences, and their children’s feelings. Finally, parents attempt to generate memories for their children and families through activities.

### 2.2 Design theme

The design topics determined based on the interview contents are divided into “Robot identity” and “Activities with robots.” Robot identity consists of two concepts. The first is “Big bro.” It stimulates the child’s curiosity and is also a friend who plays and grows with the child. The child learns cultural rules and habits by replicating Big bro’s behavior. The second concept is “Family Messenger.” It is a social robot that functions as a messenger between children and parents. Robots can understand children’s feelings, express these to their parents, and convey the parents’ love in their children’s language.

“Activities with robots” are divided into three concepts. The first is “Play for growth.” It is designed such that children can participate freely and enjoy various games. The second concept is “DIY play.” It implies the development of play using various senses such as sound, sight, and touch by utilizing objects commonly available at home. The third is “Heartfelt conversation.” Here, children gain self-understanding by talking regarding their feelings through conversation activities with robots. Additionally, children could develop empathy for others by practicing comprehending the emotions of characters in stories.

### 2.3 Developing activities

**FIGURE 1:**
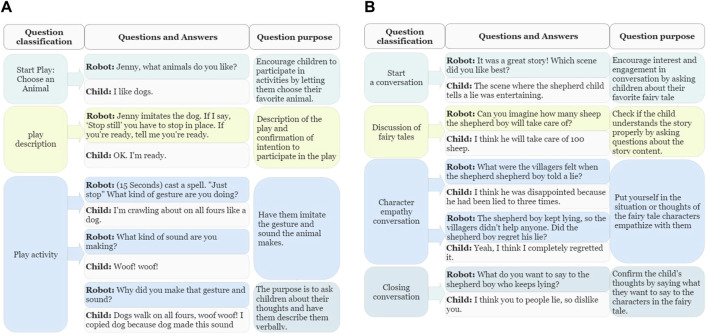
**(A)** Is an activity in the category of “Communication and verb expression” among robot play activities, and the activity name is <Enchanted Animals>. **(B)** Is an activity in the category of “Fairy Tale conversation” among robot conversation activities, and the activity name is <The Shepherd Boy and the Wolf>.

Home robot-based children’s activity services include psychomotor therapy-based play and conversation. An example activity is shown in Figure 1.

#### 2.3.1 Play activities

Play activities are related to children’s movements. These were developed by referring to widely acknowledged psychological movements. Psychological movements have been developed theoretically by Kiphard of Germany. These are mainly based on physical experience and physical movement. It is an educational and therapeutic concept that pursues the holistic development of children through creative and autonomous play and movement experiences (Kiphard, 1994).

Psychological movements constitute an opportunity for gaining sensory and motor experiences through the body. It provides physical experiences, material experiences, and social experiences (Zimmer, 1999).

Physical experience refers to physical perception, physical expression and possibility experience, and sensory experience. This study was composed of the following two categories: large and small muscles development, and communication and verb expression. Material experience refers to addressing spatial and physical situations in the surrounding world and exploration–experimental learning through movement. This study was composed of the cognition, perception, and thinking categories. Finally, social experience implies communicating, collaborating, and playing with others. In this study, it was composed of the sociality and emotion categories. A total of 52 play activities were developed: 12 for communication and verbal expression, 12 for sociality/emotion, 15 for cognition and perception and thinking, and 13 for large and small muscle development. The core goals of the four categories of play activities and one play in the category are described below:

**Communication and verbal expression** activities aim to improve children’s language skills by helping them express their thoughts, opinions, and feelings. In <Enchanted Animals>, the robot asks children to select their favorite animal and express that animal’s sound or action. After a while, the robot says, “Stop still.” And then asks, “What kind of gesture are you making?” and “What kind of sound are you making?”. These play activities help children improve their communication skills and verbal expression while describing and explaining their favorite animals.

**Sociality and emotion** activities could help children learn to care for others and collaborate. These activities include games with robots, children, and children’s friend. In <Guiding a Blindfolded Friend>, the robot asks the children to walk around the house with a blindfolded friend. The friend selects a target place, and the child blindfolds the friend and escorts him/her there. When the child arrives at the target place, the robot asks the child, “What color do you see there?”, “What is on the left?”, and “What is on the floor?” When asked by the robot, the child tells his/her friend regarding the objects around him/her. The goal of the play activities is for children to learn how to collaborate with others by helping their blindfolded friends.

**Cognition, perception, and thinking** activities aim to improve children’s problem-solving skills. In <Fisherman, How Deep Is the Water?>, the robots say, “Let’s make an imaginary river in the room with books.” The child makes an imaginary river using books. He/she moves from side to side in the river according to the robot’s instructions. Then, the robot indicates various situations during the process of the child crossing an imaginary river. “How can I cross the water?” and “The river is getting deeper. What should I do?” The robot asks the child to imagine the embarrassing situation and then, asks for an approach to address it. The goal of the play activity is for children to contemplate regarding various situations.

**Large and small muscle development** activities could help children improve their large and small muscles, body movement, and balance. In <Carrying Grain Bags>, children balance their bodies while carrying grain bags to the target place. Children fill socks with beans or grains to make grain bags. The robot asks the child to reach a target place at a distance from his/her home. The child is instructed to bring a grain bag to the target place. The robot indicates body parts such as the child’s shoulder, head, arm, and back of the hand on the way to the target place. The child moves the grain bag to the body part that the robot specifies. It is an activity wherein the child arrives at the target place without dropping the grain bag placed on the body part. This play activity helps develop children’s overall body balance.

#### 2.3.2 Conversation activities

Conversation activities were developed by referring to the Nuri course. It is a regular Korean course provided to children^1^. The Nuri process is divided into physical exercise and health, communication, social relationships, art experience, and nature exploration. Among these categories, only communication and social relationships were chosen because these are categories in which children can use robots at home to improve their skills through conversation alone, without the need for an external environment or additional supplies. In addition, when developing the various types of conversations in each category, KID KID’S^2^, GENIE KIDS^3^, Aesop’s Fables, and Grimm’s Fairy Tales were referenced.

The communication area is aimed at improving the communication skills and imagination necessary for children’s daily lives. This study was composed of the Role Play and Fairy Tale categories. The social relationship area is aimed at improving the ability to understand and respect oneself and cohabit with others. The study was composed of the social skills and problem-solving categories. A total of 71 conversation activities were developed: 11 role plays, 20 fairy tale conversations, 20 social skills, and 20 problem-solving. The core goals of the four categories of play activities and one play in the category are described below in detail:

**Role play** is an activity that helps children visualize themselves as different beings and express their thoughts, feelings, and inner thoughts. In <Wizard>, the participating children become wizards with the capability to travel at any time and to any place they wish. Robots ask when and where the child wishes to travel, who can help him/her, the steps to be adopted, and who would make him/her happy. For example, a question can be “If you could go back to a happy period in the past, which would that be, Jenny?” or “Where do you wish to travel, Jenny?”. The goal of the conversation activities is to make the child impersonate an imaginary being that can achieve anything it wishes and to induce him/her to speak his/her thoughts and feelings honestly through various questions.

**Fairy tale conversation** is an exercise in enhancing children’s emotional empathy by listening to fairy tales and asking questions regarding the characters. <The Shepherd Boy and the Wolf> involves a fairy tale of a shepherd boy who deceives villagers three times saying that a wolf has appeared. The robot begins the conversation after narrating a fairy tale to the child. First, the robot asks the child regarding his favorite scene in the fairy tale. The robot then asks the child regarding each character’s situation, e.g., “What did the villagers feel when the shepherd boy told a lie?” and “Would the shepherd boy regret his lie?”. Robots provide questions for children to contemplate regarding the character’s situation and emotion. The goal of the conversation activities is to develop children’s capability to understand the thoughts and feelings of others by placing them in the situation of characters in fairy tales.

**Social skills** conversations are intended to teach children social rules. These activities require the use of QR cards with situational images. Children show the robot a QR code and start a conversation based on the image. The <Get in Line> image shows a child passing by another individual without waiting. The robot first asks the child a question such as “What did the child in this card do that was inappropriate?”, “What happens if individuals break lines?”, or “How would other individuals feel without waiting in line?”. The robot hears the child and explains the appropriate social rules. The children can first understand situations expressed visually through QR cards and social situations and learn social rules that they were unaware of earlier.

**Problem-solving** conversations constitute an activity that help children contemplate approaches to addressing difficult social situations. In <I’m upset because I heard something unpleasant>, the robot says to the child, “I’m very upset because I heard something unpleasant from my friend today.”, “Jenny, have you ever been upset because you heard something unpleasant?”, and “How do you react, when you hear something unpleasant from a friend?”. The robot tells the child to think for themselves and develop a solution. The goal of the conversation activity is to enable children to address it and realize it by discussing regarding uncomfortable events after hearing unpleasant words.

## 3 Design of child–robot interaction

We determined that robots aimed at developing children’s social emotions need to form desirable social relationships with children. This is because conversations with partners with whom they have good rapport can be the basis for many desirable social outcomes such as highemotional quality interactions (Baker et al., 2020), increased self-disclosure (Lakin et al., 2003), and improved favorability (Vacharkulksemsuk et al., 2012).

### 3.1 Rapport

#### 3.1.1 Robot identification and self-disclosure conversation

When a robot shares a background story containing a robot’s story with a child (Darlinget al., 2015), it facilitates the child in understanding the robot and accepting it (Kory et al., 2019). Informative background stories or self-disclosure have an important role in building relationships. This is because these enable one to identify who the other individual is and to predict how that individual would behave (Markus and Nurius, 1986). In this study, the background story of the robot includes the story of revealing personal information such as the background, hobby, preference, and capability (Kory et al., 2019).

**FIGURE 2:**
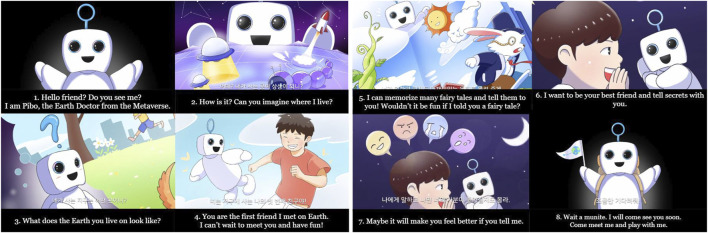
These are some of the videos that contain the identity of the robot. It contains the contents of the robot’s self-introduction, its function, its role, and its wishes. In the video, the robot concludes the video by saying that it should meet the child soon.

To form rapport between children and social robots, a video containing the identity of social robots was produced. The robot identity video is approximately 2 min 30 s long. Figure 2 displays a part of the video. Robots are designed to be perceived as social beings. Parents and children learn the identity of the robot in advance through the video before meeting it. This forms an indirect rapport with expectations regarding the robot. The content of the identity video of the social robot is as follows. The name of the social robot is PIBO. It is an Earth doctor who lives in the Metaverse. PIBO can move freely in the Metaverse, where everything that is visualized can be realized. PIBO learns regarding Earth. Earth has four seasons and various plants and animals. PIBO has no friends on Earth. The child it would meet soon would be its first friend. PIBO wishes to experience the four seasons of Earth with the child. Because PIBO knows many of the children’s favorite stories, it can narrate these whenever the child wishes. PIBO wishes to be a dear friend who shares secrets with the child. The child can narrate funny, difficult, and upsetting stories to PIBO whenever he/she wishes. PIBO states that talking to the child regarding what is upsetting him/her would help him/her feel better. It ends the video by saying that it would come meet the child soon.

Self-disclosure of social robots has been observed to affect the way children perceive and relate to robots (Gallego et al., 2019). When aiming for long-term relationship formation in robot–child interactions, self-disclosure is one of the recommended robot behaviors (Leite et al., 2014). Recent studies have revealed that robots attract children’s attention and arouse their curiosity by asking them various questions (van et al., 2022). Children tend to disclose more personal information in intimate relationships (Rotenberg and Mann, 1986). The mutual understanding formed through this sharing of personal information is known to have a positive effect on the generation of rapport between robots and children.

Self-disclosure exposing personal information is highly important for early rapport formation. After the robot arrives at the home, the first activity is a self-introduction conversation with the child. The robot asks the child’s name and age and is happy that they finally met. It asks the child what color he/she likes, and immediately modifies the color of its LED eye. The instantaneous implementation of the child’s response can enhance his/her focus. The personal information that the child conveys to the robot during the self-introduction conversation is stored as data and used for future conversations with the robot.

### 3.2 Intimacy

#### 3.2.1 Name-calling and respect response

The robot calling the child’s name is an effective strategy for inducing children’s participation and activating interactions. When the robot called the child’s name, the child responded positively with significant joy and interacted with the robot more frequently (Neumann, 2020). In other studies, children counted the number of times a robot called their name. Children with relatively larger numbers of name-calls proudly inform their less-called friends that the robot appears to like them more (Kanda et al., 2004).

The robot is designed to call the child’s name frequently during the activities. This induces the child to perceive that the robot cares about him/her (Kanda et al., 2007). In this study, the robot frequently called the child’s name and included it when asking questions regarding the child’s thoughts, experiences, and feelings. This helped maintain positive relationships between the children and robots. For example, in <Don’t do something your friend doesn’t like>, the image card question is asked, and this is followed by the child’s thoughts. “What behavior does Jenny dislike?”, “Has your friend ever done something to her that she dislikes?”, and “How would Jenny feel if her friend continues to behave like that?”. The robot shows an attitude of listening and understanding the child’s thoughts.

Responsiveness implies that the interactive partner responds appropriately to the other individual’s behavior (Davis and Perkowitz, 1979). Increasing the responsiveness (Ahmad et al., 2017) of the robot and adjusting the degree of expressive power during the activities of the child and robot tended to stimulate intimacy between them (Yasumatsu et al., 2017).

In this study, during a conversation with a child, the robot maintained an empathetic attitude without assessing whether the child’s answers are correct or not. The children’s answers are classified as positive/negative/neutral based on Bidirectional Encoder Representations from Transformers (BERT). The positive answer is “yes, good, right.” Moreover, the child’s opinion in the answer is also included as a positive. The negative answer is “no.” The neutral answer is “I don’t know.” When there is no response, the previous question is repeated two times to elicit opinions on the absence of children and whether they wish to continue the conversation. When a child answers or responds positively to an opinion, the robot responds with “You think so, I think so too.” When a child responds negatively or neutrally, they maintain a positive relationship with the child by responding with “Really?”, “Hmm,” and “It’s okay if you don’t know. I can tell you!” The robot’s response was designed for the robot to be perceived as friendly and comfortable by the children. With these reactions, children can freely express their thoughts and feelings to the robot.

#### 3.2.2 Kinetics and touch

When social robots make human-like gestures, they become more intimate and interested (Xu, K., 2019). Social robots with physical shapes can induce children’s movements to support their active psychological and physical development (Kim et al., 2023). Additionally, when interacting with social robots, children appear to engage socially as if these are playmates (Martínez et al., 2018). Robot kinetics include robot gestures, head or body movements, posture, eye color, and screen display. These aid in communicating with children. Robots can express various emotions to children through kinetics. These can express positivity, understanding, praise, empathy, etc., in response to a child’s response. These engage children in play activities and interact with them through various nonverbal cues such as movement, sound effects, and displays. Robots use kinetics when instructing or recommending to a child, such as questioning, recommendation, praising, asking a child’s opinion such as explaining, waiting, etc. To prevent the repetition of the robot’s kinetics, the robot’s length of utterance was designed by dividing it into short and long sentences.

The physical interactions between children and robots affect the intimacy (Hieida et al., 2014). The touch induced by social robots includes the reinforcement of positive emotions and attention orientation, concentration of attention, and improvement of performance (Clements and Tracy, 1977; Feldman et al., 2003). Touching is the most direct means to influence intimacy formation (Cramer et al., 2009; Nie et al., 2012). When a child touches the forehead of a robot with a touch sensor two times, the robot is ready to begin its activities. The activity begins with the child touching his forehead two times and saying, “Let’s play together.” At the beginning of all the activities designed for the child to contact the robot, physical touch such as petting or hugging increases the robot’s intimacy with individuals. This enhances long-term friendship and trust.

## 4 Field test

### 4.1 Field test process

This study was conducted with 50 children aged five–seven years (born between 2017 and 2019). Of the total 50 participants, 30 are boys and 20 are girls. The average age of 50 children is 6.3 years (SD = 0.78). There are 10 people born in 2019, 15 people born in 2018, and 25 people born in 2017. They were recruited through online social communities. The field test was conducted at the homes of the children and their parents. The supplies required for the field test were transported to the homes of the participants before the field test began.

The field test was conducted on a date convenient for the parents. KakaoTalk was used to communicate with the parents and the research team. It is a widely known online messenger in Korea, like Facebook Messenger and WhatsApp. KakaoTalk enabled the research team to identify and resolve problems that occurred during the field test at home. For example, a robot broke down during the field test. The parents contacted the research team through KakaoTalk. Then, the research team retrieved the robot and replaced it with another unit to complete the field test. The field test was conducted over 4 days (5 days including the day of robot delivery). The actual activity involving children and the robot was conducted over 4 days. The research team recommended that parents complete all activities within 4 days of the robot’s delivery to their home. The field test was conducted in the children’s playroom or living room, which is a familiar and comfortable space for them. The parents participated in and observed all the activities with their children and the robot.

### 4.2 Field test materials

The field test materials included a robot, a robot charger, a Wi-Fi router, a wireless microphone, a QR card, and an experiment participation consent form. The robot requires a network connection to run the service. To facilitate participant network installation, the research team obtained the home network information from the participants and installed it in the robot. Additionally, a Wi-Fi router was provided in case of difficulty connecting to the internet. The research team provided the parents with a consent form for explaining the experimental process and verified their participation in the experiment. The parents returned all the products necessary for the experiment to the research team upon the completion of the experiment.

Circulus’ PIBO robot is being used to provide our service. The social robot weighs 2.2 kg and has dimensions of 250 (w)× 120 (d) × 395 (h)/mm H. It has 10 DOF (two each for the neck, shoulder, wrist, and ankle). The controller uses Raspberry Pi 4.0. Circulus OS 1 (an operating system based on Linux) forms the basis of the robot creates the basis. A 3400 mAh Li-ion (DC 12.6 V) battery supplies power. A full charge requires 2 h, and usage requires 2–5 h.

### 4.3 Field test schedule

The children participated in play and conversation activities with the robot for 4 days. The research team selected from the developed activities to be performed in the field test. The activities to be conducted in the field test were selected based on whether these could be conducted using the materials conveniently accessible at home. In addition, the activities were selected such that the activity topics did not overlap. In the activity schedule for the field test, a child and the parents conduct the activities according to the date as shown in Table 1. Details of the robot activities listed in the Field test schedule can be found in the Supplementary Appendix.

**TABLE 1:** The activity schedule for the field test performed with a child and parents.

Day	1 Day	2 Day	3 Day	4 Day
Activity Schedule	Self-introduction conversation	I end up using bad words	Cover your mouth and cough	Recommended activities
	Shepherd boy and wolf	Dancing ghost	Road made of tissue paper	Recommended activities
	Big and strong animal	Body instrument that produces sound	Moving a grain bag	Farewell greeting

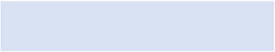
Conversation activity 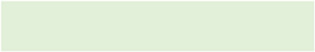
Play activity 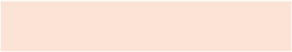
Meeting activity.

All the activities begin with a command. The command is required for the robot to execute the activity. The first self-introduction conversation starts when the child says, “Hello, nice to meet you!” to the robot. The command to start the farewell activity at the end of the fourth day of the experiment is “It’s time to say goodbye.” The command to start all the other play and conversation activities is “Let’s play.”

At the end of each activity, the robot directly asks the child regarding his/her satisfaction with the activity and receives feedback. The satisfaction score is calculated by assigning 0.5, −0.5, and −0.25 points for positive, negative, and neutral responses, respectively. The child’s satisfaction score is stored in a database and used to determine the next recommended activity. On the fourth day of the activity schedule, the robot recommends activities based on the child’s preference evaluation data and conducts the play and conversation activities. For example, if a child provides the highest score to gross/fine motor play activities among the play activities, a random play activity is highly likely to be recommended from among gross/fine motor activities.

### 4.4 Field test evaluation method

After the field tests were completed, the home service activities and robot interaction were evaluated. The evaluation method included both quantitative and qualitative evaluation.

The Godspeed scale was used for the quantitative evaluation (Bartneck, Kulic, Croft & Zohbi, 2009). The Godspeed assessment administered in this study was rated by the parents who completed the test with the child. Parents watched both robot and activities. The purpose was to measure the degree of anthropomorphism and likeability of social robots. The Godspeed scale was developed to measure robot anthropomorphism, activity, likeability, intelligence, and safety. It is used as an HRI evaluation index in many robot studies. The contents of the questionnaire are shown in Table 2. The questionnaire responses were scored using a Likert five-point scale to the side that was perceived as closer to the two of these.

**TABLE 2:** Godspeed survey items.

Godspeed			
Anthropo-morphism	Q1. Fake or Natural	Likability	Q13. Unfriendly or Friendly
	Q2. Machinelike or Humanlike		Q14. Unkind or Kind
	Q3. Unconscious or Conscious		Q15. Unpleasant or Pleasant
	Q4. Artificial or Lifelike		Q16. Awful or Nice
	Q5. Moving rigidly or Moving elegantly	Perceived Intelligence	Q17. Incompetent or Competent
Animacy	Q6. Dead or Alive		Q18. Ignorant or Knowledgeable
	Q7. Stagnant or Lively		Q19. Irresponsible or Responsible
	Q8. Mechanical or Organic		Q20. Unintelligent or Intelligent
	Q9. Artificial or Likelike		Q21. Foolish or Sensible
	Q10. Inert or Interactive	Perceived Safety	Q22. Anxious or Relaxed
	Q11. Apathetic or Responsive		Q23. Agitated or Calm
Likability	Q12. Dislike or Like		Q24. Quiescent or Surprised

In addition, a Customer Satisfaction Score (CSAT) evaluation was conducted to evaluate the satisfaction of the home service.^4^ CSAT is a measurement metric that is commonly used as a key performance indicator of customer service and product quality. The service satisfaction questionnaire items are shown in Table 3. These were evaluated using a five-point Likert scale.

**TABLE 3:** Customer Satisfaction (CSAT) assessment items.

Interview questions	
Parents	Q1. Were you overall satisfied with the play and conversation activities with the robot and the child?
	Q2. Would you like to participate in robot testing again?
Children	Q3. Did you have fun playing with robots?

The qualitative evaluation is a subjective evaluation conducted by parents through a questionnaire on service activity and robot interaction. The contents of the questionnaire are shown in Table 4.

**TABLE 4:** Home service and robot interaction survey items.

Interview questions		
Home Robot Based Activity	Play	Q1. Which play with the robot did the child enjoy the most?
		Q2. What is the reason?
	Conversation	Q3. Which conversation with the robot did the child enjoy the most?
		Q4. What is the reason?
Child-Robot Interaction	Interaction	Q5. What do you think the child's interaction with the robot was like?
		Q6. What is the reason?

## 5 Result

### 5.1 Quantitative analysis

#### 5.1.1 Analysis of godspeed

**FIGURE 3:**
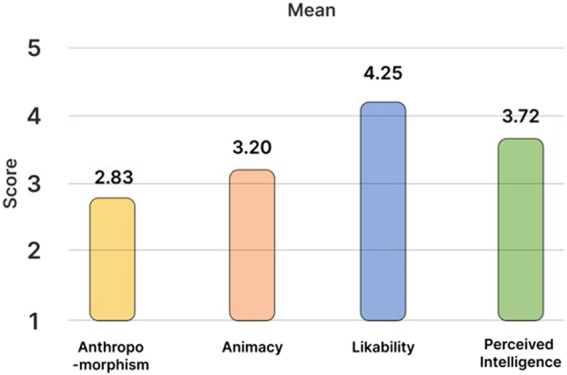
Godspeed survey results.

The godspeed results are shown in Figure 3. Godspeed’s Anthropomorphism item enables us to measure the perception of human-like behaviors and characteristics in robots. The Animacy item enables us to measure how realistic the robot’s behavior appears. The Likability item enables us to measure how friendly and attractive it is while interacting. Perceived Intelligence can measure the perception of the robot’s capability and level of knowledge. Through Perceived Safety, we measure the perceived risk or safety while interacting with the robot.

After the field test, the internal consistency was measured using Cronbach’s alpha scale in the Godspeed survey response (Bartneck et al., 2009). The survey response results were analyzed only for Anthropomorphism, Animacy, Likability, and Perceived Intelligence (Cronbach’s alpha = 0.90) with Cronbach’s alpha scores of at least 0.7. The user’s perception of the robot that provides the home social robot service developed in this study is shown in Figure 3. It received the lowest evaluation score of 2.83 points (SD = 1.16) for anthropomorphism and a score of 3.20 points (SD = 1.08) for Animacy. It obtained the highest evaluation score of 4.25 points (SD = 0.79) for Likability and a score of 3.72 points (SD = 0.94) for Perceived Intelligence (the second highest after Likability).

The Godspeed survey results reveal that it received the highest score for Likability. The children may have displayed high favorability by identifying the robot’s friendly tone and child-like voice as being more peer-friendly. It can also be assumed that the parents provided a high score to Perceived Intelligence because of the interaction aspect of robots (wherein it elicits the child’s participation in activities and responds in various manners according to the child’s words) Meanwhile, the scores were relatively low for Anthropomorphism and Animacy. In addition, although non-verbal interactions were adopted to convey emotions and situations, the robot was generally fixed in a place during the activity. Moreover, it can be interpreted that it did not receive high scores for Anthropomorphism and Animacy because it did not move large distances or continuously owing to the limited motion freedom.

#### 5.1.2 Analysis of CSAT

The satisfaction with the activity service was evaluated by parents after completing the 4-day field test. The questions and results are shown in Table 5. The research team asked parents if they were satisfied with the conversations and play activities of their children and the robots. Herein, 80% of the parents responded that they were satisfied with the service provided by the robot. In addition, 44 out of 50 parents (88%) stated their desire to participate in the robot test again. In the case of the children, the question was modified to “Did you have fun with the robot?” considering the likelihood of difficulty in understanding the word “satisfaction.” Herein, 91% of the children stated that they had fun. Thus, it was verified that the robot-based home service designed by the research team received positive reviews from both parents and children.

**TABLE 5:** Customer Satisfaction (CSAT) assessment questions and results.

Interview questions		Results
Parents	Q1. Were you overall satisfied with the play and conversation activities with the robot and the child?	80%
	Q2. Would you like to participate in robot testing again?	88%
Children	Q3. Did you have fun playing with robots?	91%

### 5.2 Qualitative analysis

#### 5.2.1 Evaluation of activities

**FIGURE 4:**
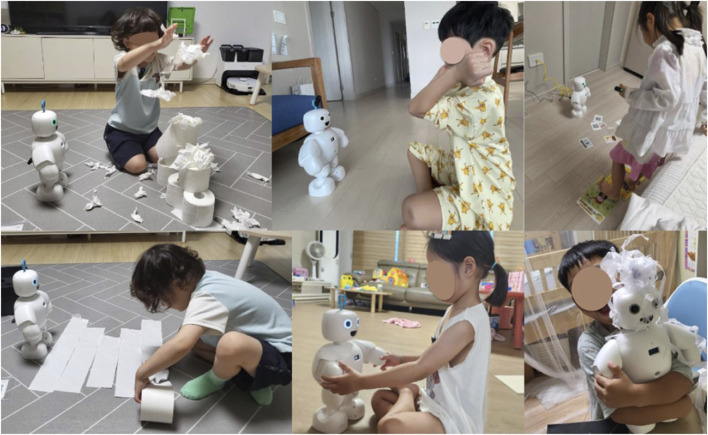
This is an image of a play activity with a robot. The photographs of the activity were received with the consent of the parents.

**FIGURE 5:**
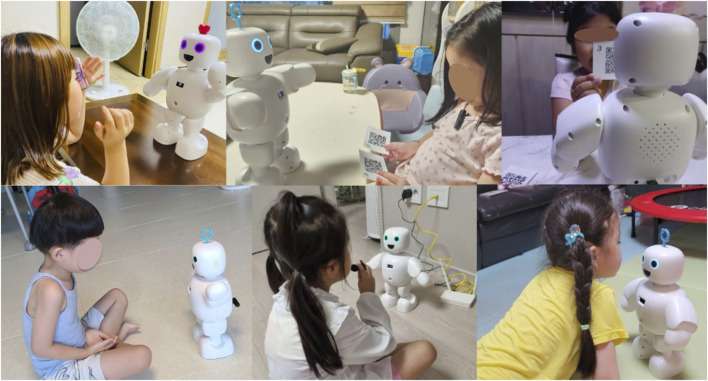
This is an image of a conversation activity with a robot.

The child’s activities with the robot at home can be seen in Figures 4, 5. These photos were taken by parents who watched the activities. Parents were asked regarding the activities that their children enjoyed the most with the robot play activities. Many parents provided different opinions. Only one duplicate answer is shown here. We have summarized the contents of the interviews. Similar responses have been combined to create themes and organize them.

**Suitability of play activities:**

“Playing activities were not excessively difficult or straightforward for the children. So, these were suitable.”

“Play activities were appropriate for the age of the child.”

“The play activities varied every day, and I cannot provide play activities of this variety at home.”

“The children enjoyed playing with the creative and engaging play activities.”

“The play activities were sufficiently short to maintain the children’s interest.”

**Physical activity:**

“My child enjoyed the variety of physical activities.”

“The child actively enjoyed the activity. He/she even replicated the robot’s movements.”

“<Moving a Grain Bag> helped the child to accurately identify body parts.”

“In <Dancing Ghost>, the child delighted in dancing to the music played by the robot.”

“The children had the most fun in the <Dancing Ghost> activity because it stimulated their imagination with the theme of ghosts.”

“The child’s mood was positively influenced after dancing.”

**Motivating active participation:**

“The robot is waiting for the child to complete his/her preparations for the play activity, it is motivating the child to participate actively.”

“When performing an activity that requires tissues, the robot said, “We need tissues. Please find it in the house.” The child found the tissues on his/her own and continued the activity. I think this helps the child's spontaneity.”

“The child appeared to laugh and focus substantially on activities such as <Dancing Ghost> and Body Instrument that Produces Sound> that the child did on his/her own.”d

Combining the parents’ opinions, they determined that the play activities were appropriate for their children’s level. Parents were also impressed with the significant variety in play activities. Parents stated that their children enjoyed activities that encouraged them to dance or move to songs such as *Dancing Ghost* and *Moving a Grain Bag* the most. Parents also perceived it to be a considerate form of play for their children wherein the robot asked the child, “*Tell me if you’re ready”* rather than unilaterally conducting the play activity. Finally, parents considered that playing with robots on their own could enhance their children’s spontaneity.

Parents were asked regarding their children’s favorite conversation activity with the robots. The interview contents were organized if topics overlapped.

**Suitability of conversation activities:**

“I am pleased with the robot’s interactions with my children. It appears that the conversation activities were intended for children.”

“Questions such as “Do you wish to continue playing at night?” were appropriate for a child.”

“I consider that the robot and child were friendly. It continued to talk, and asked questions fit the child's level.”

**Emotional conversation:**

“I was captivated by the fact that the robot asked questions regarding experiences and emotions to help the child think and to lead the child’s thoughts.”

“I was touched when the robot asked the child who was important to them, and the child rep lied that it is mom.”

“I heard events related to my child's kindergarten that I was unaware of by listening in on the conversations between the robot and child.”

**Friendly conversation:**

“It’s nice to be able to communicate two-way. A conversation between a robot and child is like conversing with a real person.”

“The self-introduction conversation was highly similar to my child meeting a friend!”

“The child liked a fairy tale narrated by a robot in a kind manner and wished to hear another one.”

“The child responded more positively to the robot’s questions than to their parents’ questions.”

**QR card conversation:**

“My child enjoyed using image etiquette QR card!”

“Seeing the situation with a card helps you better understand the conversation with the robot. Moreover, it is good for your child to concentrate.”

“During the etiquette QR card conversation, there was a content that said, “I’m coughing while covering my mouth.” The child saw the etiquette card and talked to the robot first, saying he was coughing.”

“The robot and the child were in the middle of a QR card conversation with a friend who had robbed the toy. The robot asked the child, “Do you have any friends like that?”. The child told the robot the name of his friend at his kindergarten and talked to it. It appeared that the robot and child were discussing regarding a child's concerns.”

Parents determined that the conversation activity was appropriate for their child’s level. They stated that the self-introduction conversation resembled a child and robot meeting a new friend and that it was good for children to express their thoughts and feelings through conversations with robots. Additionally, they considered it remarkable to be informed regarding their child’s thoughts that they were unaware of earlier. In addition, the QR card with the image of social situations stimulated the child’s curiosity and helped them understand the social situation necessary for their conversation with the robot. In addition, the robot asked questions by comparing these to the child’s immediate situation, as shown in the image. It was highly remarkable that it understood the child’s deep feelings and provided guidance on how to respond appropriately, such as counselling.

#### 5.2.2 Evaluation of robot interaction

We asked for opinions regarding the child–robot interaction designed by the research team. The following feedback was received.

**Name-calling:**

“My child was waiting for when the robot would come home, and he kept asking about the robot’s name.”

“My child liked it because the robot called my child’s name like a friend.”

“When the robot called my child’s name, it was like talking to my child’s real friend.”

**Respect response:**

“It was impressive that the robot responded kindly and sympathized with the child’s answer.”

“I liked the part where the robot answered, “If you don’t know, it’s okay.”

“The robot said, “You think so,” and it was surprising that the conversation with the child was smooth. It was like learning the art of conversation from a robot.”

“The robot and the child were discussing regarding the weather. When the child said he liked rainy days, the robot responded, “I like it too.” The child loved having something in common with the robot.”

“When the robot responded to a child’s question, the tone of the robot’s answer was so impressive (“oh, ho”) that people imitated it in their daily lives and enjoyed it.”

“After the conversation, the robot asked the child if it was fun. Each time, the robot answered that it was fun to talk to the child, which made the child very happy.”

**Kinetics:**

“My child always had fun imitating the robot’s kinetics. I really enjoyed watching that.”

“When the robot’s eyes turned red, the child was a little scared. Otherwise, my child was focused on the color variation in the robot’s eyes.”

**Touch:** “The child always carried the robot around the house.”

“The child really liked the robot. He even covered the robot with his favorite blanket.”

The robot interaction designed by the research team to form rapport and to enhance the intimacy between robots and children had the following characteristics. The robot provided respectful responses to the child to increase parental satisfaction. Moreover, the robot called the children’s names in a friendly manner and positively influenced the formation of rapport with children. Encouraging children to imitate the movements of a robot can be fun for children. Touching the robot to start the activity did not appear to have a direct effect on the positive rapport formation between children and robots. This is because touching the robot is limited to the role of a power button to start the activity.

## 6 Lessons learned

The purpose of this study was to develop robot-based home services to nurture social emotions in children. As a field test, home services were provided for robots, children, and parents at home for 4 days. Then, the effectiveness of the service was verified through a satisfaction evaluation and interviews with parents and children.

In the service design phase, the requirements were identified through interviews with parents and child development experts. The service theme was developed based on these interviews. The play and conversation services were developed based on psychomotor therapy techniques and the guidelines for children’s education by the Ministry of Education of Korea. In addition, based on previous research on the interaction between robots and children, a method was designed to increase the intimacy between robots and children. The research results are explained in connection with the design themes developed while designing the service and the verification results.

**Building Rapport—Big Bro:** The study identified that rapport may be developed between a child and robot via both direct and indirect encounters. Indirect interactions such as watching a video regarding the robot’s identity before meeting it can help develop children’s expectations. It is consistent with the observations that the identity of social robots (which describe their existence and characteristics) attracts children’s attention and strengthens their understanding of social robots. This, in turn, forms personal connections (Jae hee Chung, 2023). A self-introduction conversation is a conversation in which individuals reveal personal information, thoughts, and feelings to each other. It was verified in the interviews with the parents that while talking regarding himself/herself with the robot that he/she had encountered for the first time, he/she had positive feelings similar to having encountered a real friend. Based on these results, this study verified that while designing interactions between children and robots, the formation of rapport between them can be promoted through connected interactions such as an “identity video” before meeting and “meeting conversation” after it.

**Person–Family Messenger:** Robots function as a medium to help improve the relationship between parents and children. The robot-based home service is differentiated from the function-centered robot service design that has been demonstrated. It is designed with a focus on relationships with the family. The parent interview verified that parents considered it remarkable that they could learn regarding the child’s honest feelings, thoughts, and experiences in the external environment (that the parents were not aware of earlier) through the conversations between the child and robot. This is consistent with parents’ stated intention to be knowledgeable of their children’s friendships and inner feelings. Accordingly, robots are likely to contribute to parents’ overall understanding of their children’s lives as an individual and increase family bonds.

**Equal Status—Growth Play:** The play activities and conversations with the child provided by the robot are adapted to the child’s eye level. This imparts the child with the perception of playing with a friend. Children tend to express their thoughts and feelings freely to peers they consider equals (Sankaran and Badzis, 2017). The PIBO social robot used in this service is a small humanoid robot that speaks in a child’s voice. Therefore, the child accepts it as a friend. The robot’s voice imparts one with the perception that it is talking to a child’s friend. Putting social robots on equal footing with their peers could influence children’s honesty or participation in volunteer activities and provide them with positive experiences. Establishing the robot as having an equal status with the child is also considered as a crucial factor in designing the interaction between children and robots.

**Safety, Comfort: DIY Games:** This indicates that the comfortable location, person, and time should be considered when encouraging children’s active participation in services. The field test in this study was conducted in a manner wherein children interacted with the robot with their families in a familiar home environment. The home environment provides comfort and safety for children. Family members also provide trust and support for the child, which makes the child feel comfortable. When designing children’s interactions, it is important to consider the home environment and family members to create an environment in which children can participate more actively. This is a factor that enables children to participate more actively in robot activities and increase satisfaction.

Respect, consideration, empathy—Heartfelt conversations: The robot’s responsive design is aimed at respecting, caring, and empathizing with children.

In play activities, the robot added a waiting response such as “Tell me when you’re ready.” Parents expressed that the overall response of the robot made them perceive it to be waiting for and respecting their child. The robot’s response should be noncritical of the child’s response. Friendly responses should be designed so that children can speak comfortably. These polite and considerate responses from the robot substantially contributed toward increasing the intimacy between the child and robot.

**Multi-user:** For a balanced design, the needs of children (the actual users of the robot) should also be considered. Children may be incapable of fully expressing their opinions owing to the limitations of language skills. However, they would be capable of understanding their needs through observation techniques (Gardner, 2000). The developed service considers only the needs of parents. During the on-site inspection, the child was observed to be searching for the mother because he wished to play with her. Therefore, to achieve a balanced and optimal service design, the needs of two users [the buyer (parent) and consumer (child)] should be considered.

## 7 Conclusion

This study designed a home robot-based children’s activity service. A field test was conducted for 4 days at the home of the child and parents. After completing the field test, we conducted quantitative and qualitative evaluations and discussed the results. In addition, we discussed the aspects to consider while designing children’s activity services using social robots. Thus, this study would aid service designers and engineers who design services for children through social robots.

## Data Availability

The original contributions presented in the study are included in the article/Supplementary Material, further inquiries can be directed to the corresponding authors.

## Footnotes

1
https://i-nuri.go.kr/ebook/20200731huv/index.html

2
https://www.kidkids.net/

3
https://www.genikids.com/

4
https://www.qualtrics.com/kr/experience-management/customer/what-is-csat/
