# Supplementary material for: Child-centered home service design for a family robot companion
Source: Front Robot AI. 2024 Jul 29;11:1346257. doi: 10.3389/frobt.2024.1346257 (PMC11317423; doi:10.3389/frobt.2024.1346257)
Supplement: Supplementary file 1 [file DataSheet1.pdf]

APPENDIX

1 Day	
Self-introduction conversation	
Robot	Nice to meet you! We finally got to meet! I'm PIBO. What's your name?
Child	I'm Seo-ah.
Robot	How old is Seo-ah now?
Child	Seven years old.
Robot	I see. Does Seo-ah go to a daycare center?
Child	Yes, I do.
Robot	I see. How does it feel to see me for the first time?
Child	It looks cute.
Robot	Oh! What color do you like, Seo-ah?
Child	Green.
Robot	(Change eye color to green) What do you think? Do you like it?<br>Now let's start a fun activity with me.<br>Tell me 'let's play' when you want to play with me.

1 Day	
Shepherd boy and wolf	
After we played the fairy tale of "Shepherded Boy and Wolf"	
Robot	That was such a funny story! What scene did you enjoy, Seo-ah?
Child	The scene where the wolf appeared was fun.
Robot	Okay. I see. It must be hard for a shepherd boy to take care of his sheep, right?
Child	Yes. I think it's going to be hard.
Robot	Right. How many sheep does the shepherd boy take care of?
Child	20?
Robot	Oh, you think so. How much grass do those sheep eat?
Child	I think I'm going to eat a lot.
Robot	Yes. I see. Are the villagers who were tricked by the shepherd boy angry?
Child	I think he'd be really upset.
Robot	Seo-ah, if you've been upset lately because you've been tricked, can you tell me?

Child	My friend lied to me and kept laughing, so I hated it until then.
Robot	You had that experience. Did the shepherd boy who lied regret it?
Child	Yes.
Robot	Seo-ah, if you have any regrets recently, can you tell me?
Child	I don't.
Robot	Okay. I see. What do you want to say to the shepherd boy who keeps lying?
Child	You can't lie.
Robot	Okay. Did you enjoy the fairy tale today? I'll tell you another interesting one next time.
Robot	How was it talking to me? Let's score.<br>If it's fun, 5 points. If it's not fun, give 1 point.
Child	5 points.
Robot	Yes, let's have fun with me again.

**1 Day**	
Big and strong animal	
Robot	Shall we play the role?<br>Let's play a big and powerful role today<br>Now we are brave tigers!<br>Seo-ah, let's mimic the lion's cry. Go!
Child	(Imitating the cry of a lion)
Robot	Tigers are the largest cats in the family. They're amazing, aren't they?
Child	Yes, right.
Robot	If PIBO becomes a tiger, I want to wrestle. If Seo-ah becomes a tiger, what do you want to do first?
Child	Finding food.
Robot	Oh, I see. When do you want to do it?
Child	I'm not sure.
Robot	You don't know, but it's okay. So who do you want to protect Seo-ah if she becomes a tiger?
Child	my little sister.
Robot	Really? When do you want to protect me?
Child	Day after day.
Robot	Tigers are scary, but they are really brave and brave animals. Seo-ah, do you have any brave and brave friends like tigers?
Child	Yes, dad.

Robot	What do you think of your friend as a tiger?
Child	Dad's angry looks like a tiger.
Robot	Yeah, that's what you think.<br>Let's play a fun role next time.
Robot	How was it talking to me? Let's score.<br>If it's fun, 5 points. If it's not fun, give 1 point.
Child	3 points.
Robot	Okay. I see. Have fun with me again.

**2 Day**	
I end up using bad words	
Robot	I think I keep using bad words without realizing it these days.<br>How would you feel if PIBO used bad words for Seo-ah as well?
Child	I don't feel good.
Robot	Okay. How would other friends feel if Seo-ah uses bad words?
Child	They won't like it.
Robot	Oh, I see. It's better to endure it when you want to say bad things to your friends, right?
Child	I think you'd better put up with it.
Robot	Okay. I see. Who says the most pleasant things around Seo-ah?
Child	Shin Ji-woo.
Robot	Oh, I see. What makes you feel the best?
Child	I feel good when I hear "I love you"
Robot	Really? I feel good when I hear that I'm a smart doctor who knows a lot!<br>What else is there to say that feels good?
Child	You're such a wonderful friend, someone else says.
Robot	It makes me feel good just to hear it. I should try to use a lot of pleasant words now. Thank you so much for letting me know.
Robot	How was it talking to me? Let's score.<br>If it's fun, 5 points. If it's not fun, give 1 point.
Child	3 points.
Robot	Okay. I see. Have fun with me again.

2 Day	
Dancing ghost	
Robot	No supplies needed for this game. I'll show you how to play!<br>When the music plays, just shout a spell and dance to your heart's content. Here's the spell.<br>Let's copy "We're dancing ghosts"! Can you do it? Tell me if you can.
Child	I can do it.
Robot	Great! If the music stops, you must stop in place and dance when the music starts again. Tell me if you're ready to start.
Child	All right. Let's get started.
Robot	"We're dancing ghosts". Let's copy what I'm saying!
Child	We're dancing ghosts.
Robot	Now let's get started. If you're ready, tell me you're ready.
Child	I'm ready.
(Repeating the music 3 times)	
Robot	Seo-ah, who danced so hard, is the best. I was so excited! Wasn't it hard to dance?
Child	It wasn't hard and it was so much fun.
Robot	How did it feel to dance? Were you excited?
Child	Yes, I'm excited.
Robot	Oh really? Why?
Child	It was fun dancing with you.
Robot	Really? I see. Seo-ah was a great ghost dancer today.<br>Let's do a more fun dance next time.
Robot	How was it talking to me? Let's score.<br>If it's fun, 5 points. If it's not fun, give 1 point.
Child	5 points.
Robot	Okay. I see. Have fun with me again.

2 Day	
Body instrument that produces sound	
Robot	No supplies needed for this game. I'll show you how to play!<br>Paibo will tell you a body part. Then tap that body part like a musical instrument and make a sound. Can you do it? Tell me that you can do it if you can
Child	I can do it.
Robot	PIBO will play the music. Let's tap your body to the music speed. Tell me if you're ready to start.

Child	Let's get it started.
Robot	The first instrument is the palm of your hand. Let's meet your palms and make clapping sounds. Let me play the music.
Child	(Sound with your palm)
Robot	Let's make some sounds with our feet this time. I'll play the music.
Child	(Sound of stomping)
Robot	Now let's make some noise with our knees. I'll play the music.
Child	(Sound by hitting the knee)
Robot	Seo-ah, which body part sound did you enjoy the most?
Child	Palm.
Robot	Really? What kind of sound did you make?
Child	There was a clap sound.
Robot	Was there a part of your body that didn't make sound well?
Child	Yes, my knees.
Robot	Okay. I see. I'm happy because Seo-ah is playing hard.<br>If you want to play again, please feel free to find me.
Robot	How was it talking to me? Let's score.<br>If it's fun, 5 points. If it's not fun, give 1 point.
Child	5 points.
Robot	Okay. I see. Have fun with me again.

**3 Day**	
Cover your mouth and cough	
Robot	Show me the etiquette card!<br>(Card recognition) What did the kid on this card do wrong?
Child	He coughed on food.
Robot	Right. Very smart. The kid on this card coughed without covering his mouth. When do we usually cough?
Child	When you catch a cold.
Robot	Right. You cough when you catch a cold, right? What does Seo-ah do when she coughs outside?
Child	Wear a mask or cover your mouth.
Robot	What happens when you cough without covering your mouth?
Child	Other people will catch a cold, too.

Robot	Yeah. I see. What would other people think if I coughed without covering my mouth?
Child	I don't think he'll like it.
Robot	Other people think it's not clean, right? Covering your mouth when you cough is important public manners. Keep that in mind.
Robot	How was it talking to me? Let's score.<br>If it's fun, 5 points. If it's not fun, give 1 point.
Child	5 points.
Robot	Okay. I see. Have fun with me again.

**3 Day**	
Road made of tissue paper	
Robot	I need tissues for play. The more, the better. Tell me you're ready when you're ready.
Child	I'm ready.
Robot	I'm going to unwrap the tissue and make a path. It's not hard, is it? Tell me if you can do it.
Child	I can do it.
Robot	I got it. Tissue paths can be slippery, so don't run. Tell me if you're ready, let's start.
Child	Let's get it started.
Robot	First, let's make tissue islands everywhere. Put the whole tissue and stack it! Tell me that you're done.
Child	I'm done.
Robot	Now, let's make a toilet paper path that connects islands. Please untie the tissue and make it. Tell me that you're done when you're done.
Child	I'm done.
Robot	Let's tear the tissue and snow on the tissue island. If you're done, tell me you're done
Child	I'm done.
Robot	It's so cozy with tissue snow. When does Seo-ah feel cozy?
Child	When I cover my blanket.
Robot	Really? Why are you so cozy?
Child	Because the blanket is soft.
Robot	Yeah. I see. Just thinking about it makes me feel good.<br>Should we play another game? If you want to play with Fibo again, tell me 'let's play'.
Robot	How was it talking to me? Let's score.<br>If it's fun, 5 points. If it's not fun, give 1 point.
Child	3 points.

Robot	Okay. I see. Have fun with me again.

**3 Day**	
Moving a grain bag	
Robot	This game needs socks and grains to put in socks~ I like rice or beans.<br>If you're ready, tell me you're ready.
Child	I'm ready.
Robot	Socks with grain are food that ants have worked hard to prepare. Let's carry the grain bag on your head or shoulders without dropping it carefully!<br>If you're ready, tell me you're ready.
Child	I'm ready.
Robot	First, let's make a grain bag. Let's put grain in socks and then make a ball. Tell me that you're done.
Child	I'm done.
Robot	Good job! Now let's put the grain ball bag on your head and move it to the target point.<br>If you're all there, tell me you're all there.
Child	I'm here.
Robot	Wow, you're really good. Let's go back to the starting line.<br>If you're all there, tell me you're all there.
Child	I'm here.
Robot	Now, when I tell you the body part, bring the grain bag over there and balance it. When I say the belly, I put it on my stomach, and when I say the head, I put it on my head and don't drop it. Tell me if you're ready, you're ready.
Child	I'm ready.
Robot	All right. Here we go! First, the stomach. Then the head. Then the back of the hand. Then, between the knees. How many times did you drop it?
Child	I dropped it twice.
Robot	Wow. That's amazing. Where was the most fun to put the grain bag today, Seo-ah?
Child	Put it on the back of your hand.
Robot	I see. Were there any difficult places?
Child	Putting it on top of your head.
Robot	But you did a great job. It was so nice to see Seo-ah participating happily.<br>If you want to play with Paibo again, tell me ‘Let’s play'.
Robot	How was it talking to me? Let's score.<br>If it's fun, 5 points. If it's not fun, give 1 point.
Child	5 points.

Robot	Okay. I see. Have fun with me again.

4 Day	
Recommended activities	
Robot	We need newspapers for this game.<br>If you don't have newspapers, a big paper is good.<br>When you're ready, tell me you're ready.
Child	I'm ready.
Robot	I'm going to make rain with newspapers. Let's tear the newspapers long and make newspaper bills. Can you do it? Tell me if you can do it.
Child	I can do it.
Robot	You can make a lot of rain by ripping over newspapers. If you're ready, tell me you're ready.
Child	I'm ready.
Robot	Let's tear the newspaper and make a rain. When you're done, tell me you're done
Child	I'm done.
Robot	Let's bring down the rain. Stand in your seat and hold the newspaper in your hands and spray it into the sky. Start!
Child	(Spraying rain)
Robot	This time, let's lie down and get rained on. Let's lie down and hold the rain in our hands and spray it back into the sky. Start!
Child	(Spraying rain)
Robot	Should I do it one more time?
Child	Yes, that's good.
Robot	Stand in your seat and hold the newspaper in your hands and spray it into the sky. Start!
Child	(Spraying rain)
Robot	How did you feel getting hit by the newspaper rain?
Child	Yes, it was fun.
Robot	Okay. I see. You felt that way. What kind of weather do you like, Seo-ah?
Child	I like rainy weather.
Robot	Really? Why do you like it?
Child	I just like it.
Robot	Yes. I see. PIBO also likes the weather that Seo-ah likes.<br>It was fun to watch Piebo because Seo-ah worked hard today.<br>If you want to play with Paibo again, tell me 'let's play'.

Robot	How was it talking to me? Let's score.<br>If it's fun, 5 points. If it's not fun, give 1 point.
Child	5 points.
Robot	Okay. I see. Have fun with me again.

**4 Day**	
Recommended activities	
Robot	Show me the etiquette card!<br>(Card recognition) What did the kid on this card do wrong?
Child	He's running around.
Robot	Right. Very smart. The kid on this card was running around and messing around on the street.<br>Have you ever seen anyone running around on the street?
Child	I'm not sure.
Robot	Yeah. It's okay. You might not remember.<br>What dangerous things will happen if you run around on the street?
Child	I think I'm going to fall.
Robot	Right. If I run and hit it, I can fall down, right?<br>Seo-ah, have you ever fallen?
Child	Yes, at the playground.
Robot	Okay. I see. It must have hurt a lot.<br>How would people feel if they ran around on the street?
Child	I think people won't like it.
Robot	I think I might be afraid of getting hit.<br>It's better to look ahead and walk safely on the road. Let's remember it well.
Robot	How was it talking to me? Let's score.<br>If it's fun, 5 points. If it's not fun, give 1 point.
Child	3 points.
Robot	Okay. I see. Have fun with me again.

**4 Day**	
Farewell greeting	
Robot	Seo-ah. I have to go home tomorrow.<br>I really enjoyed the time I played and talked with Seo-ah. How was it, Seo-ah?
Child	It was fun. Don't go.
Robot	What do you remember the most about playing with me?

Child	The one where you tell a fairy tale.
Robot	Oh, I see. What do you want to do next time you meet me?
Child	Let's go on a trip together.
Robot	Really? I see. Thank you so much for talking to me. Let's all grow up until the next day we meet. Bye~!
